# Loss of PTEN expression is associated with PI3K pathway-dependent metabolic reprogramming in hepatocellular carcinoma

**DOI:** 10.1186/s12964-020-00622-w

**Published:** 2020-08-24

**Authors:** Chuanzong Zhao, Ben Wang, Enyu Liu, Zongli Zhang

**Affiliations:** 1 Department of General Surgery, Qilu Hospital of Shandong University, No. 107, Wenhua West Road, Lixia District, Jinan, 250012 Shandong Province P. R. China; 2 Key Laboratory for Experimental Teratology of the Ministry of Education and Department of Pathology, School of Medicine, Shandong University, Jinan, 250012 P. R. China

**Keywords:** Hepatocellular carcinoma, Metabolic reprogramming, Warburg effect, PTEN, PI3K pathway, Glucose uptake, Lactate production

## Abstract

**Background:**

Metabolic reprogramming, in which energetic metabolism changes from oxidative phosphorylation to glycolysis, is well-accepted as a hallmark of cancers including hepatocellular carcinoma (HCC). A growing body of evidence suggests the involvement of oncogenes and tumor suppressor genes in the control of metabolic reprogramming. In this study, we attempt to investigate whether loss of PTEN, a recognized tumor suppressor, drives metabolic reprogramming of HCC.

**Methods:**

Cancerous liver tissues were surgically resected from 128 HCC patients, with 43 adjacent noncancerous liver tissues as control. Aerobic glycolysis (Warburg effect) was reflected by measurements of glucose uptake and lactate production, mitochondrial membrane potential collapse was observed by JC-1 staining, glycolytic rate and mitochondrial respiration were evaluated by determining glycolytic proton efflux rate (glycoPER) and oxygen consumption rate (OCR) in cultured human HHCC cells.

**Results:**

Reciprocal expression of PTEN and PI3K was determined in cancer liver tissues. Overexpression of PTEN suppressed the Warburg effect, as evidenced by reductions in glucose uptake and lactate production, maintenance of mitochondrial function, and transformation of energetic metabolism from glycolysis to oxidative phosphorylation in cultured PTEN-negative HHCC cells. Importantly, 740 Y-P, a PI3K agonist that leads to activation of the PI3K pathway, partially abrogated the function of PTEN and reprogramed glucose metabolism in cultured HHCC cells.

**Conclusions:**

The discovery that loss of PTEN allows the tumor metabolic program has been a major advance in understanding the carcinogenesis of HCC.

Video abstract

**Graphical abstract:**

Graphic abstract showing that loss of PTEN regulates the tumor metabolic program in hepatocellular carcinoma. Loss of PTEN leads to activation of the PI3K pathway enhances the Warburg effect, thereby promoting the development of hepatocellular carcinoma. 
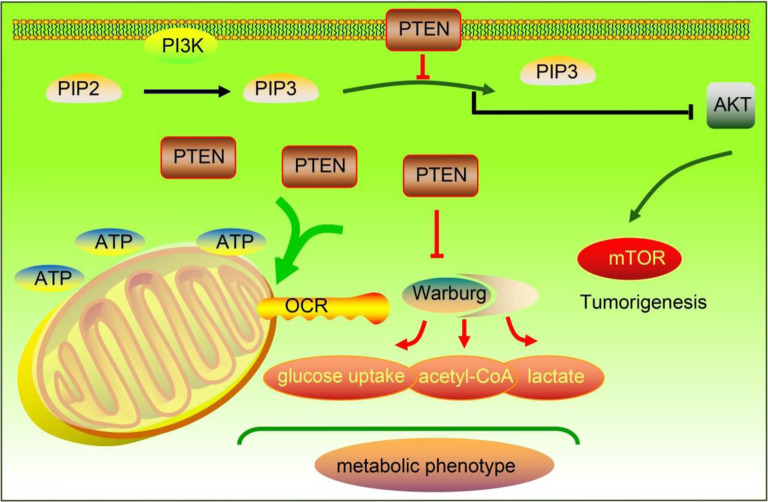

## Background

Hepatocellular carcinoma (HCC) represents a malignant tumor predominantly arising in the setting of cirrhosis, causing an estimated one million deaths on a global scale in 2030 [1]. According to the Global Cancer Statistics 2018, the mortality rate of HCC is only second to lung cancer in males by gender stratification [2]. Most of HCC cases at their initial diagnosis have reached the advanced stage, which results in poor patient survival [3]. An enhanced understanding of the mechanism underlying HCC pathogenesis, recurrence and metastasis is required to achieve early diagnosis and to further develop more effective treatment modalities.

Cancer cells preferentially consume glucose and glutamine to fuels uncontrolled cell proliferation and process intermediate metabolites for lipid and protein synthesis, which drives tumorigenesis [4]. Metabolic reprogramming is an emerging hallmark in liver cancer cells where energetic metabolism shifts from oxidative phosphorylation to aerobic glycolysis, so-called Warburg effect [5]. Abnormal expression of oncogenes or tumor suppressor genes could regulate the metabolic network to aid in cancer cell growth and survival in adverse microenvironment. Well-known oncogenes such as Kras and Akt directly control the activation of metabolic pathways in proliferating cells, including cancer cells [6, 7]. Besides, tumor suppressor genes are involved in the control of the metabolic switch in cancers [8], such as p53 [9]. However, the understanding of reciprocal regulations between anabolic pathways and cellular pathways are still in its infancy, and more extensive investigations are required.

The PI3K/Akt/mTOR pathway is commonly activated in human cancers and contributes to oncogenic transformation including proliferation, survival, and metabolic reprogramming [10]. Phosphatase and tensin homolog (PTEN) is the second most frequently mutated gene in cancers including HCC [11] and encodes a phosphatase protein that returns the PI3K/Akt/mTOR pathway to its inactivated state [12]. Cancer cells lacking PTEN expression manifest a glycolytic phenotype, which is the Warburg effect [13]. Emerging evidence supports PTEN accumulation reduced hypoxia-induced Akt phosphorylation, thereby repressing glycolysis in cancer cells [14]. However, whether cell metabolic reprogramming is one of the consequences of PTEN deficiency in HCC remains to be investigated. In the present study, we investigated the effects of PTEN on HCC cell metabolism, in a bid to highlight a framework for the metabolic function of tumor suppressor PTEN in HCC.

## Materials and methods

### Ethics statement

The study protocol received the approval from the Ethics committee of Qilu Hospital of Shandong University and is in strict accordance with the *Declaration of Helsinki*. All participants signed informed consent documentation prior to sample collection. Animal studies were performed with the approval from the institutional animal care and use committee of Shandong University, with extensive efforts made to ensure minimal suffering of the animals used during the study.

### Human specimens

Cancerous liver tissues were surgically resected from 128 HCC patients who had been diagnosed with HCC at Qilu Hospital of Shandong University from July 2011 to July 2014, with 43 adjacent noncancerous liver tissues (absence of cancer cells) as controls. Clinical information of patients was available from medical record department, which included age, gender, tumor classification, tumor stage, tumor size, smoking history, and metastasis (presence or absence). No patients received anti-tumor therapy prior to tissue sample collection. After surgery, all patients were followed up 60 months until November 2019.

### Immunohistochemical staining

Deparaffined and hydrated liver tissue sections were immunostained for PTEN and PI3K using rabbit anti-human antibody against PTEN (ab170941, 1:200, Abcam, Cambridge, UK) and PI3K (AF6242, 1:100, Affinity, Jiangsu, China). Visualization was performed using 3,3-diaminobezidine (DAB; ST033, Whiga Biosmart Co., Ltd., Guangzhou, China). Five high-power fields of view were randomly captured for each replicate and 100 cells in total were counted in each field using a microscope. Staining intensities were scored as 0 and 1 (negative), 2 (moderate) and 3 (positive). Ten fields were randomly selected at 200 × magnification and observed. The staining intensities were divided into four grades (0–3 points): 0 indicates invisible positive staining; 1 indicates faint staining; 2 indicates moderate staining; 3 indicates strong positive staining. The proportion of positive cells was also scored from 0 to 3 points, in which 0 indicates tumor cells without positive staining, 1 indicates positive cells less than 20%, 2 indicates 20–50% of positive cells, and 3 indicates more than 50% positive cells. Final staining scores, consisting of intensity and proportion scores, 0–1 were defined as negative/weak, 2–4 as positive and 6–9 as strong positive.

### Cell culture, lentiviral transduction, and agonist treatment

Human normal hepatocyte HL-7702 (L^− 02^) and four immortalized HCC cell lines HHCC, HepG2, Hep3B, and SMMC7721 purchased from American Type Culture Collection (Manassas, VA, USA) were cultured in RPMI1640 medium (Thermo Fisher Scientific, Rockford, IL, USA) supplemented with 10% fetal bovine serum (FBS) and 100 μg/mL streptomycin and 100 U/mL penicillin in a 5% CO_2_ incubator with saturated humidity (95%) at 37 °C. Cultures of HHCC were transduced for 72 h with a lentiviral vector (MOI: 10) encoding full-length human PTEN (Shanghai GeneChem, China). The infected HHCC cells were selected by 2-week puromycin (1.5 μg/mL) treatment, and puromycin-resistant colonies were picked, expanded, and analyzed. 740 Y-P (CAS1236188–16-1, MedChemExpress Company, Beijing, China), a PI3K agonist, was added into HHCC cells for activation of the PI3K pathway.

### Western blot analysis and antibodies

HHCC cells were lysed using protease inhibitor-contained RIPA buffer (Beyotime Biotechnology Co., Ltd., Shanghai, China) for protein extraction. After SDS-PAGE analysis, the protein was transferred onto PVDF membranes and probed with the following primary antibodies (Abcam): anti-PCNA (ab18197), anti-Ki67 (ab16667), anti-PTEN (ab170941), anti-PI3K (ab70912), anti-p-PI3K (ab32089), anti-Akt (ab8805), anti-p-Akt (ab81283), and anti-mTOR (ab2732). Immunoblots were exposed to horseradish peroxidase-coupled goat anti-rabbit immunoglobulin (ab205719, Abcam) followed by enhanced chemiluminescence detection reagents (EMD Millipore, USA). Target protein bands were quantified using ImageJ software, and GAPDH was sued for normalization.

### Cell cycle analysis and cell apoptosis assays

Cell nuclei were stained with propidium iodide (PI) using the kit (FXP031–100, Beijing 4A Biotech Co., Ltd., China) and analyzed by flow cytometry (FACScan®; BD Biosciences) equipped with CellQuest software (BD Biosciences) to examine the distribution of cells throughout G0/G1, S, and G2/M phases of the cell cycle. For cell apoptosis assays, PI was used in conjunction with Annexin V (FXP018–100 kit, Beijing 4A Biotech Co., Ltd., China) to determine if cells were viable, apoptotic, or necrotic by flow cytometry.

### Cell proliferation assay

Cells were seeded into 24-well plates and stained with EdU solution for 2 h. EdU-stained cells were fixed with 4% paraformaldehyde for 15 min, followed by 20 min of incubation with PBS containing 0.5% Triton X-100. The nuclei were stained using DAPI in the dark and observed under a fluorescence microscope (FM-600, Shanghai Pudan Optical Instrument Co. Ltd., Shanghai, China). Six to ten fields were randomly captured to count the number of EdU-stained cells (proliferating cells) and DAPI-stained nuclei.

### Cell migration assay

Cells were seeded into the 6-well plate with 5 × 10^5^ cells/well. A thin scratch (10 μL) was created along the center of each well with a sterile pipette tip (the width of each scratch was the same). To evaluate wound closure, six fields were selected, and the cells were photographed at 0 h and 24 h after incubation with serum-free medium. The cells in the wound area were counted and analyzed by counting software.

### Cell invasion assays

Cell invasion assays were carried out using transwell chamber assays (BD Biosciences, San Jose, CA, USA) according to instruction provided by the manufacturer. Briefly, the cells were resuspended into 1 × 10^5^ cells/mL using the serum-free medium and placed into the upper chambers coated with Matrigel (BD Biosciences, Bedford, MD, USA) that had been diluted with serum-free DMEM. After 24 h of incubation at 37 °C, the cells that transferred to the lower chamber containing 5% FBS-supplemented DMEM as a chemoattractant were stained with 0.1% toluidine blue and counted in five random fields per well using an inverted optical microscope (Carl Zeiss, Inc., Oberkochen, Germany).

### Tumorigenicity assays of human HCC cells in nude mice

A total of 36 Specific pathogen-free (SPF)-conditioned nude mice (aged 6–8 weeks) were purchased the Beijing HFK Bioscience Co., Ltd. (Beijing, China). The mice were housed individually in specific pathogen-free (SPF) animal laboratory at 22–25 °C with 60–65% humidity, a 12-h light/dark cycle and ad libitum access to food and water. After 1 week of adaptive feeding, the experiment was conducted. HHCC cells with lentiviral transduction of PTEN expression vector or empty vector were resuspended at a concentration of 1 × 10^7^ cells/mL, and then 200 μL of cell suspension was subcutaneously inoculated into each mouse. Tumor growth was observed and recorded every week, with the growth curve plotted. HCC xenograft tumors were collected and subject to RT-qPCR and immunohistochemical staining.

### Assessment of Warburg effect

Warburg effect describes an energy shift from mitochondrial oxidative phosphorylation to aerobic glycolysis and is evaluated by measurements of glucose uptake, acetyl-coenzyme A (acetyl-CoA) synthesis, and lactate production. In cancer cells, pyruvate is converted to lactate in spite of the presence of oxygen. Glucose uptake, pyruvate and lactate production were determined using Glucose Uptake Colorimetric Assay kit (ab136955), Pyruvate Colorimetric/Fluorometric Assay Kit (K609–100), and Lactate Assay Kit II (K607–100), respectively, following the protocols provided by manufacturers (Biovision, K686–100, Milpitas, CA, USA). All determinations were done in triplicate.

### JC-1 staining

JC-1, a cationic dye, was used to evaluate mitochondrial membrane potential (MMP) collapse. We evaluated the extent of MMP damage using the kit (#C2006, Beyotime Biotechnology, Wuhan, China) following the protocols provided by manufacturers. In brief, HHCC cells were incubated with 100 μL JC-1 dye solution at 37 °C for 30 min. Then JC-1-stained cells were washed with serum-free medium and analyzed with a fluorescence microscope. Red stains indicate JC-1 aggregates in intact mitochondria, and green stains indicate JC-1 monomer in apoptotic cells with depolarization of MMP. The ratio of red/green fluorescence intensity was obtained to evaluate MMP.

### Measurements of extracellular acidification rate (ECAR) and oxygen consumption rate (OCR)

The ECAR and OCR were examined using Seahorse XF Glycolytic Rate Assay Kit (103344–100, Seahorse Bioscience, North Billerica, MA, USA) and Seahorse XF Mitochondrial Respiration Assay Kit (103260–100, Seahorse Bioscience) as per kits’ protocols, respectively and analyzed with the Seahorse XFe 24 Extracellular Flux Analyzer (Seahorse Bioscience) as previously described [15].

### Statistical analysis

For statistical comparisons, the unpaired Student t test, a one-way analysis of variance (ANOVA) with Tukey’s test, and repeated measurements ANOVA with Bonferroni corrections, and the chi-square test were performed as appropriate. Spearman’s correlation coefficient was used for statistical correlation. Survival curves were plotted using Kaplan-Meier’s method, and statistical differences were identified by a log-rank test. Cox’s proportional hazards model was employed to identify the independent factors. All statistical analyses were performed with SPSS 21.0 software (IBM, Armonk, NY, USA), with two-tailed *p* < 0.05 as a level of statistical significance.

## Results

### PTEN is downregulated in HCC and correlates with poor prognosis of HCC

**Fig. 1:**
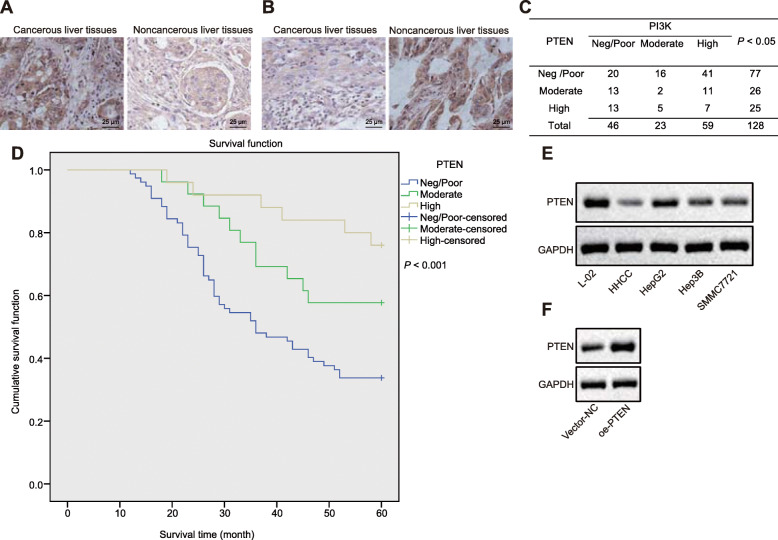
PTEN is downregulated in HCC and its loss predicts worse prognosis in HCC patients. **a** and **b**, Immunocytochemistry staining of PTEN (**a**) and PI3K (**b**) proteins in cancerous liver tissues (*n* = 128) and adjacent noncancerous liver tissues (*n* = 43) (× 400). * *p* < 0.05 compared with adjacent noncancerous liver tissues by unpaired *t*-test. **c**, Spearman’s correlation analysis of PTEN expression with PI3K expression in HCC tissues (*n* = 128). **d**, Kaplan-Meier curves are plotted to show overall survival of 128 HCC patients according to PTEN expression. **e**, Representative Western blots of PTEN protein in L-02, HepG2, Hep3B, SMMC7721, and HHCC cell lines, normalized to GAPDH. * *p* < 0.05 compared with L-02 cell lines. **f**, Verification of HHCC cells with lentiviral transduction of PTEN by Western blot analysis, normalized to GAPDH. HHCC cells were transduced with a lentiviral vector encoding full-length human PTEN (oe-PTEN), with those cells infected with a lentiviral vector harboring empty expression vector as controls (vector-NC). * *p* < 0.05 compared with vector-NC. Data are shown as mean ± standard deviation of three technical replicates

Loss of PTEN has been a common molecular event during carcinogenesis. To investigate oncogenic and prognostic performance of PTEN and its underlying mechanism in HCC, we first performed immunohistochemical staining to examine the expression of PTEN and PI3K in 128 cancerous liver tissues and 43 adjacent noncancerous liver tissues. The results showed weaker immunohistochemical staining for PTEN protein (Fig. 1a, Table 1) but stronger immunohistochemical staining for PI3K protein (Fig. 1b, Table 2) in cancerous liver tissues than in adjacent noncancerous liver tissues. Analysis using Spearman’s correlation coefficient showed that PTEN expression was negatively correlated with PI3K expression in cancerous liver tissues (Fig. 1c). Additionally, the low expression of PTEN was associated with tumor classification and metastasis of HCC patients (*p* < 0.05, Table 3). As shown by the Kaplan-Meier curves, patients with decreased PTEN expression had shorter overall survival than those with increased PTEN expression (*p* < 0.05, Fig. 1d). Cox regression analysis showed that the low expression of PTEN was an independent risk factor for overall survival of HCC (HR: 2.952, 95%CI: 1.979–4.403, *p <* 0.05, Table 4). Next, we quantified the expression of PTEN protein in human HCC cells, HHCC, HepG2, Hep3B, SMMC7721, and normal liver cells L-02 by Western blot analysis. The results displayed a lower expression of PTEN in HCC cell lines than that in normal cells, among which HHCC cells presented the lowest PTEN expression and were thus selected for subsequent in vitro experiments (Fig. 1e). In the following experiments, we infected HHCC cells with a lentiviral vector carrying PTEN expression vector, and lentivirus-mediated overexpression of PTEN was confirmed by Western blot analysis (Fig. 1f).

**Table 1:** Expression of PTEN in cancerous liver tissues (*n* = 128) and adjacent noncancerous liver tissues (*n* = 43)

Group	N	PTEN			Positive expression rate (%)	*P*
		Poor	Moderate	High		
Adjacent Noncancerous liver tissues	43	0	8	35	100	< 0.001**
Cancerous liver tissues	128	77	26	25	39.84	

** denotes statistical significance by chi-square test

**Table 2:** Expression of PI3K in cancerous liver tissues (*n* = 128) and adjacent noncancerous liver tissues (*n* = 43)

Group	N	PI3K			Positive expression rate (%)	*p*
		Poor	Moderate	High		
Adjacent Noncancerous liver tissues	43	35	6	2	100	< 0.001**
Cancerous liver tissues	128	46	23	59	39.84	

** indicates statistical significance by chi-square test

**Table 3:** Correlation of PTEN expression with the clinicopathological characteristics of HCC patients

Variable	PTEN expression			Total (*n* = 128)	*P*
	Negative (*n* = 77)	Moderate (*n* = 26)	High (*n* = 25)		
Gender					
Male	54	19	15	88	0.553
Female	23	7	10	40	
Age					
< 60	45	16	13	74	0.776
≥ 60	32	10	12	54	
Stage					
I + II	12	21	24	57	< 0.001**
III	61	4	1	66	
Unknown	4	1	0	5	
Metastasis					
Absence	26	17	23	66	< 0.001**
Presence	47	5	1	53	
Unknown	4	4	1	9	
Tumor size					
≤ 3 cm	38	13	14	65	0.957
> 3 cm	35	11	10	56	
Unknown	4	2	1	7	

** denotes statistical significance by chi-square test

**Table 4:** Cox regression analysis showed PTEN was an independent prognostic factor for HCC

Factor	B	SE	Wald	*P*	HR	95% CI (lower-upper)
Age	−0.285	0.252	1.273	0.259	0.752	0.459–1.233
Tumor stage	0.492	0.217	5.128	0.024*	1.635	1.068–2.502
Tumor size	−0.157	0.207	0.575	0.448	0.855	0.570–1.282
Low expression of PTEN	1.001	0.479	4.378	0.036*	2.722	1.065–6.952

* denotes statistical significance

### Overexpression of PTEN inhibits HCC cell proliferation, migration, and invasion while inducing apoptosis in vitro in addition to repressing tumorigenicity in vivo

**Fig. 2:**
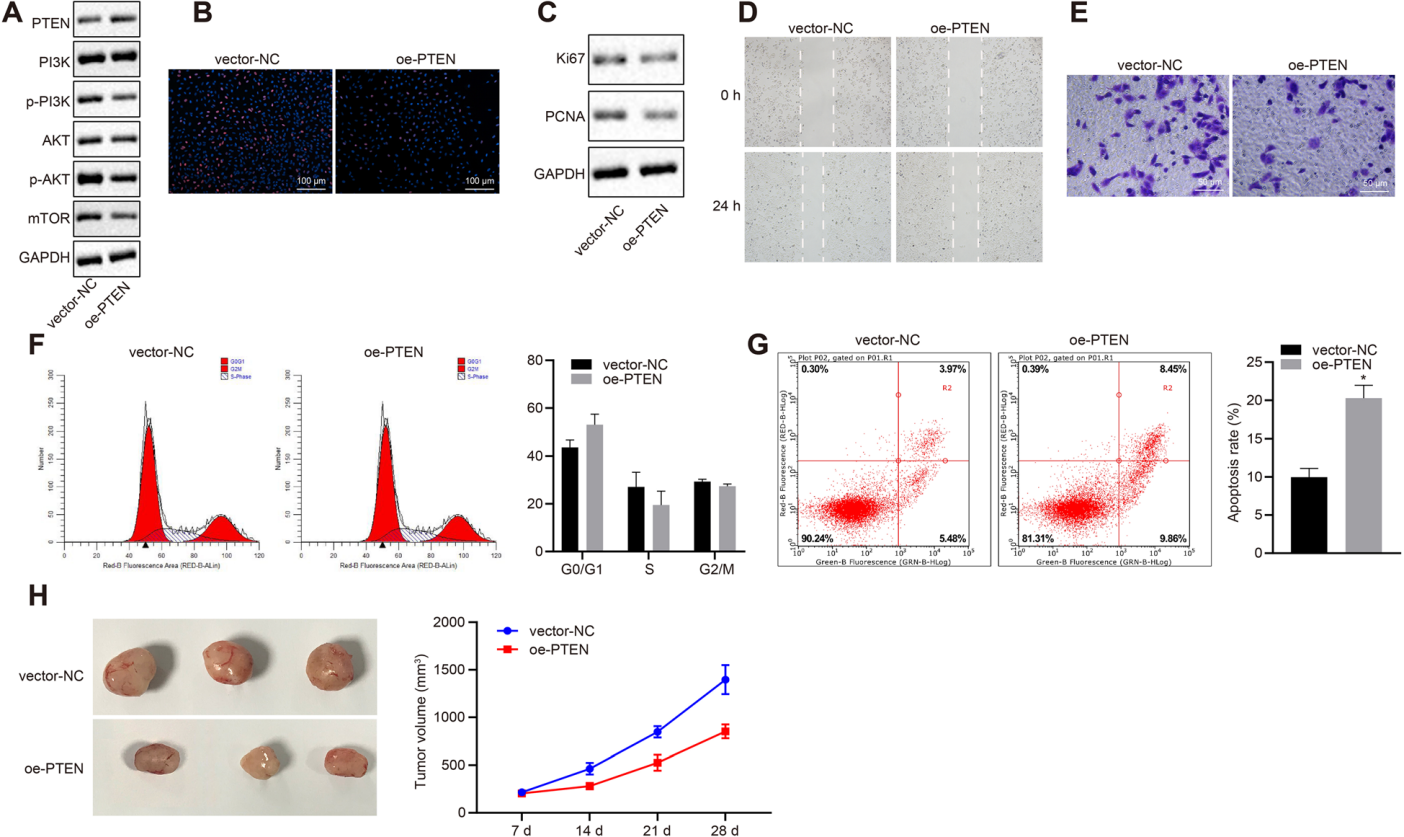
PTEN regulates the development of HCC in vitro and in vivo by inhibiting the activation of PI3K/Akt pathway. HHCC cells were transduced with a lentiviral vector encoding full-length human PTEN (oe-PTEN), with those cells infected with a lentiviral vector harboring empty expression vector as controls (vector-NC). **a**, Representative Western blots of PI3K, Akt, and mTOR proteins and their quantitation in HHCC cells, normalized to GAPDH. **b**, EdU-stained cells were captured (× 200) to reflect HHCC cell proliferation. **c**, Representative Western blots of proliferation markers Ki67 and PCNA and their quantitation in HHCC cells, normalized to GAPDH. **d**, Wound closure was monitored to measure HHCC cell migration (24 h after scratch). **e**, HHCC cells invading from Matrigel-coated the upper transwell chamber into the lower one. **f**, Flow cytometric analysis of PI staining was performed to examine the distribution of HHCC cells throughout G0/G1, S, and G2/M phases of the cell cycle. **g**, Flow cytometric analysis of Annexin V/PI double staining was performed to determine HHCC cell apoptosis. **h**, Representative HCC xenograft tumors and the growth of HCC xenograft tumor measured every 7 days in nude mice (*n* = 6). * *p* < 0.05 compared with vector-NC by unpaired *t*-test. Data are shown as mean ± standard deviation of three technical replicates

In this part, we aim to investigate the regulation and mechanism of PTEN in HCC. Western blot analysis results revealed that lentivirus-mediated overexpression of PTEN resulted in declined extent of PI3K and Akt phosphorylation with decreased protein level of mTOR, while total expression of PI3K and Akt did not differ (*p <* 0.05, Fig. 2a). To examine the contribution of PTEN to HCC development, EdU staining showed that HHCC cell proliferation was decreased following lentivirus-mediated overexpression of PTEN (*p <* 0.05, Fig. 2b). In parallel experiments, proliferation markers Ki67 and proliferating cell nuclear antigen (PCNA) were determined by Western blot analysis to reflect HHCC cell proliferation. The results showed the protein expression of Ki67 and PCNA was declined in HHCC cells following lentivirus-mediated overexpression of PTEN (*p <* 0.05, Fig. 2c). As shown in Fig. 2d, e, scratch test and transwell invasion assays demonstrated that HHCC cell migration and invasion following lentivirus-mediated overexpression of PTEN following lentivirus-mediated overexpression of PTEN following lentivirus-mediated overexpression of PTEN (*p <* 0.05). Flow cytometric analysis of PI staining and Annexin V/PI double staining showed more HHCC cells arrested at the G0/G1 phase with enhanced apoptosis in following lentivirus-mediated overexpression of PTEN (*p <* 0.05, Fig. 2f, g). Tumorigenicity assay displayed reduced tumor growth in nude mice with subcutaneous inoculation of human HHCC cells with lentivirus-mediated overexpression of PTEN (*p <* 0.05, Fig. 2h). These results unveil that PTEN inhibits HCC cell proliferation, invasion and migration while inducing their apoptosis in vitro as well as inhibiting tumorigenicity in vivo.

### Overexpression of PTEN inhibits Warburg effect and maintains mitochondrial function in HCC cells

**Fig. 3:**
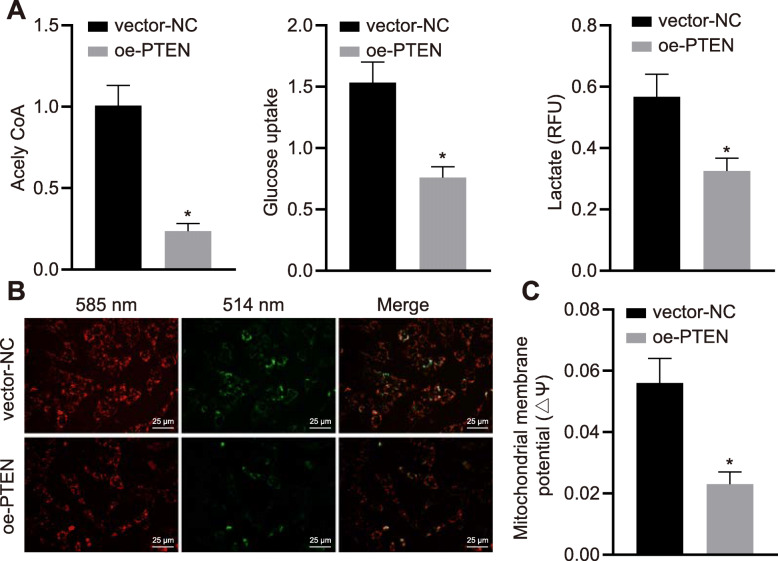
PTEN inhibits Warburg effect and maintains mitochondrial function in HCC cells. HHCC cells were transduced with a lentiviral vector encoding full-length human PTEN (oe-PTEN), with those cells infected with a lentiviral vector harboring empty expression vector as controls (vector-NC). **a**, Measurements of acetyl-CoA synthesis, glucose uptake, and lactate production in HHCC cells. **b**, JC-1 staining was performed to examine the MMP in HHCC cells (× 400). Red stains at 585 nm indicate JC-1 aggregates in intact mitochondria, and green stains at 514 nm indicate JC-1 monomer in apoptotic cells with depolarization of MMP. **c**, Ratio of red/green fluorescence intensity was calculated by flow cytometric analysis in HHCC cells. * *p* < 0.05 compared with vector-NC by unpaired *t*-test. Data are shown as mean ± standard deviation of three technical replicates

To elucidate whether loss of PTEN drives the progression of HCC, our ongoing studies were to test the effects of PTEN on metabolic phenotype, known as the Warburg effect in HCC. Warburg effect is evaluated by measuring the important indicators, including glucose uptake, glycolytic enzyme aldolase, and lactate and pyruvate production. Once PTEN was elevated in HHCC cells, glucose uptake (*p <* 0.05), lactic acid level (*p <* 0.01), and acetyl-CoA synthesis (*p <* 0.001) were declined (Fig. 3a). Also, malignant cell metabolism commonly shifts from mitochondrial respiration to aerobic glycolysis. To test the effects of PTEN on mitochondrial function of HCC cells, we performed JC-1 staining to examine the MMP in HHCC cells. Not surprisingly, overexpression of PTEN enhanced the MMP and maintained mitochondrial function of HHCC cells, as evidenced by declined JC-1 aggregates (green dots) in HHCC cells with lentivirus-mediated overexpression of PTEN (Fig. 3b). Additionally, JC-1-labled flow cytometric analysis showed that lentivirus-mediated overexpression of PTEN led to a decreased proportion of red to green fluorescence in HHCC cells (Fig. 3c). These results unveil that restoration of PTEN inhibits Warburg effect and maintains mitochondrial function in HCC cells.

### Overexpression of PTEN changed metabolic phenotype of HCC cells in vitro

**Fig. 4:**
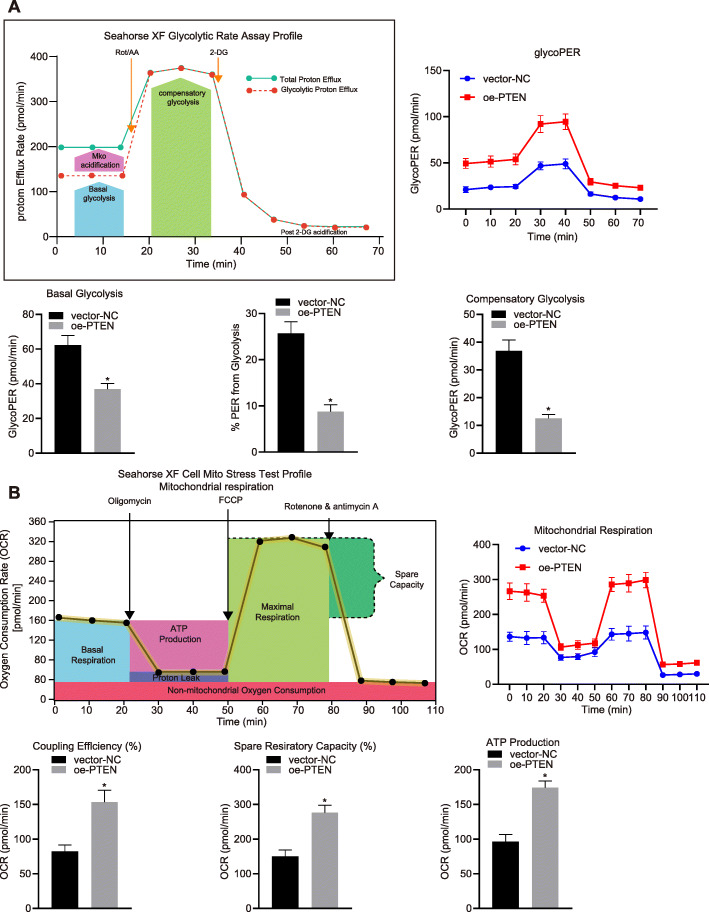
PTEN reduces ECAR and enhances OCR in HCC cells. HHCC cells were transduced with a lentiviral vector encoding full-length human PTEN (oe-PTEN), with those cells infected with a lentiviral vector harboring empty expression vector as controls (vector-NC). At first, the ECAR and OCR were measured under basal condition. Next, the ECAR was measured in the presence of rotenone plus the mitochondrial complex III inhibitor antimycin A (Rote/AA) and the glycolytic inhibitor 2-DG at indicated time points. The OCR was measured in the presence of oligomycin, the reversible inhibitor of oxidative phosphorylation FCCP (p-trifluoromethoxy carbonyl cyanide phenylhydrazone), and the Rote/AA at indicated time points. OCR is expressed as pmols/minute and ECAR as mpH/minute. **a**, ECAR under basal glycolysis, %ECAR from basal glycolysis, and the ECAR of compensatory glycolysis were measured by Seahorse Bioscience XF24 analyzer in HHCC cells. **b**, basal OCR, ATP-linked respiration, maximal OCR, and spare respiratory capacity were measured by Seahorse Bioscience XF24 analyzer in HHCC cells. Data in the upper (**a** and **b**) were compared using unpaired *t*-test and in the lower (**a** and **b**) by repeated measures ANOVA with Bonferroni corrections. * *p* < 0.05 compared with vector-NC. Data are shown as mean ± standard deviation of three technical replicates

Energetic metabolism shift from mitochondrial respiration to aerobic glycolysis often occurs in Cancer cells. We next investigate the effects of PTEN on metabolic phenotype of HCC cells by measuring the ECAR and OCR. We found that lentivirus-mediated overexpression of PTEN reduced the glycolysis rate of HCC cells, as evidenced by reduced ECAR under basal glycolysis, lower percentage of ECAR from basal glycolysis, and reduced ECAR of compensatory glycolysis in HHCC cells with lentiviral transduction of oe-PTEN compared with vector-NC (Fig. 4a). As expected, we found that lentivirus-mediated overexpression of PTEN enhanced mitochondrial respiration of HCC cells, as evidenced by enhanced basal OCR, ATP-linked respiration, maximal OCR, spare respiratory capacity in HHCC cells with lentiviral transduction of oe-PTEN compared with vector-NC (Fig. 4b). These results suggest that overexpression of PTEN leads to transformation of energetic metabolism from glycolysis to oxidative phosphorylation in vitro.

### Overexpression of PTEN suppressed HCC cell metabolic reprograming by deactivating the PI3K pathway in vitro

**Fig. 5:**
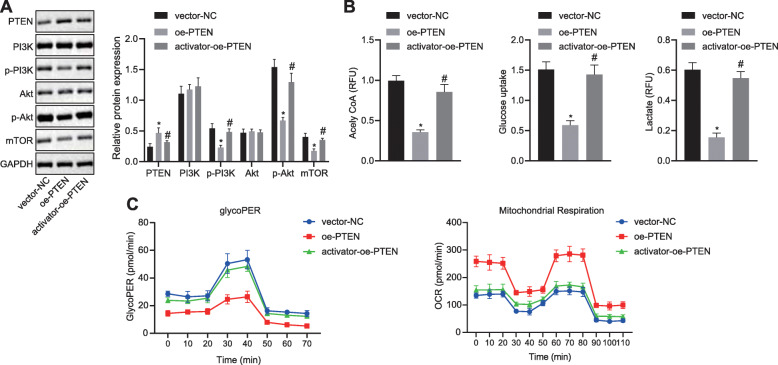
PTEN inhibits Warburg effect and maintains mitochondrial respiration instead of aerobic glycolysis by inhibiting the PI3K pathway in vitro. HHCC cells were transduced with lentiviral vector harboring PTEN (oe-PTEN) with or without the addition of 740 Y-P, a PI3K agonist (activator +oe-PTEN). HHCC cells transduced with lentiviral vector harboring empty vector were served as controls (vector-NC). **a**, Representative Western blots of Akt, PI3K, and mTOR proteins and their quantitation in HHCC cells, normalized to GAPDH. **b**, Measurements of acetyl-CoA synthesis, glucose uptake, and lactate production in HHCC cells. **c**, ECAR and OCR at indicated time points in HHCC cells. * *p* < 0.05 compared with vector-NC; # *p* < 0.05 compared with oe-PTEN. Data in (**c**) were compared by repeated measures ANOVA with Bonferroni corrections and in (**a** and **b**) by one-way ANOVA with Tukey’s test. Data are shown as mean ± standard deviation of three technical replicates

Since a negative correlation between PTEN and PI3K expression was detected in HCC tissues, we lastly studied whether the loss of PTEN reprogramed HCC cell metabolism by the activation of PI3K pathway. For this purpose, HCC cells stably expressing PTEN were treated with 740 Y-P, a PI3K agonist alone or in combination with PTEN overexpression. The extent of PI3K and Akt phosphorylation and the protein level of mTOR were enhanced in response to treatment 740 Y-P with oe-PTEN in HHCC cells, whereas the total PI3K and Akt expression remained unchanged (Fig. 5a). Additionally, treatment with both 740 Y-P and oe-PTEN was found to increase glucose uptake, acetyl-CoA synthesis, lactate production, ECAR, and decrease OCR compared to oe-PTEN treatment alone in HHCC cells (Fig. 5b, c), suggesting activation of the PI3K pathway reprogramed HCC cell metabolism even in the presence of PTEN. These results suggest that PTEN inhibits Warburg effect and maintains mitochondrial respiration instead of aerobic glycolysis by inhibiting the PI3K pathway in vitro.

## Discussion

HCC, as the 4th most frequently occurring cancer across the world, accounts for 75–85% cases of primary liver malignancies, yet the corresponding management and biomarkers are still in urgent need for this refractory disorder [16]. The current investigation was performed with the aim to unravel the functional role of an antioncogene, PTEN, in the metabolic reprogramming of HCC. Our experimental data provided solid evidence proving that the presence of elevated PTEN harbored the potential to restore mitochondrial function via inactivation of the PI3K/Akt pathway.

At the initial stage of our investigation, it was found that PTEN was expressed at low levels in HCC tissues and cell lines and it was in association with shorter overall survival of patients, staging and metastasis. PTEN has been reported to be downregulated in nearly half of the cases of HCC tissues while reduction of PTEN expression is identified as an independent biomarker for the recurrence and overall survival of HCC as it leads to reduced recurrence-free survival and overall survival of patients [17]. Concordant with our results, PTEN has been proposed as a promising biomarker of the tumor grade and disease stage in patients with HCC considering its role in counterbalancing apoptosis and proliferation [18]. Besides, accumulating evidences have suggested the participation of the activated PI3K/Akt pathway in the growth-promoting effects of PTEN in HCC [19–22]. The blockade of the PI3K/AKT/mTOR pathway can help efficiently induce apoptosis and inhibit the growth, migration, and invasion of human HCC cells [23]. Likewise, in our study, high expression of PI3K was also observed in HCC tissues and delivery of a lentiviral vector containing human PTEN exerted inhibitory effects on HCC cell malignant behaviors while inducing apoptosis by inhibiting the PI3K/Akt pathway.

In addition to diverse molecular targets developed for treatment of HCC, cancer metabolism has been attracting research attention recently as a novel mechanism to offer new perspectives of developing potential therapeutic strategies for HCC [24]. Of note, there are two pronounced metabolic pathways that have been documented as the reprogramming of cancer metabolism, i.e., mitochondrial oxidative phosphorylation and glycolysis [25, 26]. In our study, experimental data revealed that overexpressed PTEN resulted in significantly suppressed glycolysis and enhanced mitochondrial respiration to restore mitochondrial function through the inactivated PI3K/Akt pathway. The shift from mitochondrial respiration to cytosolic glycolysis of the Warburg effect has been indicated as a prelude to the occurrence of HCC [27]. Additionally, HCC cells have been allowed to acclimate the exposure to hypoxia by means of Warburg effect [28]. Warburg effect is usually characterized by fermentation from glucose to lactate and elevated glucose uptake, which are highly suggestive of altered metabolism, offering a favorable environment where cancer cells are attempting to grow, survive, proliferate, and maintain in a long term [29, 30]. Consistently, our results showed that the anti-tumor effects of elevated PTEN expression were in parallel with inhibited Warburg effect as evidenced by decreased levels of glucose uptake and lactate. Largely in agreement with our results, systemic elevation of PTEN has been demonstrated to create a metabolic state against tumor progression by negatively impacting the Warburg effect, the mechanism of which can depend on the PI3K pathway [31]. Depletion of PTEN works in tandem with the activation of the PI3K pathway in contribution to increased mitochondrial respiratory capacity and glycolysis rate in hepatocytes [32]. Similarly, upregulated PTEN has been elucidated to induce glioblastoma cell apoptosis and curb cell viability through mediation on mitochondrial dysfunction by inactivating the Akt pathway [33]. Comparably, the PTEN/PI3K/Akt axis has been unmasked to play a part in breast cancer cell apoptosis mediated by mitochondria [34], supporting the validation of our findings to be worthy of exploitation regarding its potential application value.

## Conclusion

To conclude, PTEN was demonstrated to be involved in the metabolic reprogramming during the progression of HCC that PTEN depletion acted as an independent biomarker for undesirable oncologic outcomes of patients with HCC while PTEN elevation contributed to the restoration of mitochondrial dysfunction by inhibiting the activation of the PI3K/Akt pathway. Importantly, the present investigation provides a novel therapeutic biomarker and strategy against the development of HCC. However, further experiments are still required by relating the results in tissues and cells to the human clinical setting for determination of clinical application value of the reported axis.

## Data Availability

The datasets generated/analyzed during the current study are available.

## Abbreviations

HCC: Hepatocellular carcinoma
PI3K/Akt/mTOR: Phosphatidylinositol 3-kinase/protein kinase B/mechanistic target of rapamycin
PTEN: Phosphatase and tensin homolog
RPMI: Roswell Park Memorial Institute
oe: Overexpression
NC: Negative control
glycoPER: Glycolytic proton efflux rate
OCR: Oxygen consumption rate
DAB: 3,3-diaminobezidine
FBS: fetal bovine serum
PBS: phosphate-buffered saline
DMEM: Dulbecco’s modified Eagle’s medium
GAPDH: Glyceraldehyde-3-phosphate dehydrogenase
RIPA: Radio-immunoprecipitation assay
BCA: Bicinchoninic acid
PVDF: Polyvinylidene fluoride
SDA-PAGE: Sodium dodecyl sulfate polyacrylamide gel electrophoresis
PCNA: Proliferating cell nuclear antigen
p: Phosphorylated
DAPI: 4′,6-diamidino-2-phenylindole
EdU: 5-ethynyl-2′-deoxyuridine
FITC: Fluorescein isothiocyanate
PI: Propidium iodide
SPF: Specific pathogen-free
MMP: Mitochondrial membrane potential
ECAR: Extracellular acidification rate
acetyl-CoA: Acetyl-coenzyme A
ANOVA: Analysis of variance
OS: Overall survival
HR: Hazard ratio
CI: Confidence interval

## References

[1] Villanueva A (2019). Hepatocellular carcinoma. N Engl J Med.

[2] Bray F, Ferlay J, Soerjomataram I, Siegel RL, Torre LA, Jemal A (2018). Global cancer statistics 2018: GLOBOCAN estimates of incidence and mortality worldwide for 36 cancers in 185 countries. CA Cancer J Clin.

[3] Kulik L, El-Serag HB (2019). Epidemiology and Management of Hepatocellular Carcinoma. Gastroenterology..

[4] Yang L, Venneti S, Nagrath D (2017). Glutaminolysis: a Hallmark of cancer metabolism. Annu Rev Biomed Eng.

[5] Nakagawa H, Hayata Y, Kawamura S, Yamada T, Fujiwara N, Koike K (2018). Lipid metabolic reprogramming in hepatocellular carcinoma. Cancers (Basel).

[6] Ying H, Kimmelman AC, Lyssiotis CA, Hua S, Chu GC, Fletcher-Sananikone E (2012). Oncogenic Kras maintains pancreatic tumors through regulation of anabolic glucose metabolism. Cell..

[7] Li Q, Zhao Q, Zhang J, Zhou L, Zhang W, Chua B (2019). The protein phosphatase 1 complex is a direct target of AKT that links insulin signaling to hepatic glycogen deposition. Cell Rep.

[8] Levine AJ, Puzio-Kuter AM (2010). The control of the metabolic switch in cancers by oncogenes and tumor suppressor genes. Science..

[9] Liu J, Zhang C, Hu W, Feng Z (2015). Tumor suppressor p53 and its mutants in cancer metabolism. Cancer Lett.

[10] Hoxhaj G, Manning BD (2020). The PI3K-AKT network at the interface of oncogenic signalling and cancer metabolism. Nat Rev Cancer.

[11] Peyrou M, Bourgoin L, Poher AL, Altirriba J, Maeder C, Caillon A (2015). Hepatic PTEN deficiency improves muscle insulin sensitivity and decreases adiposity in mice. J Hepatol.

[12] Yehia L, Keel E, Eng C (2020). The clinical Spectrum of PTEN mutations. Annu Rev Med.

[13] Cordero-Espinoza L, Hagen T (2013). Increased concentrations of fructose 2,6-bisphosphate contribute to the Warburg effect in phosphatase and tensin homolog (PTEN)-deficient cells. J Biol Chem.

[14] Chen F, Zhuang M, Zhong C, Peng J, Wang X, Li J (2015). Baicalein reverses hypoxia-induced 5-FU resistance in gastric cancer AGS cells through suppression of glycolysis and the PTEN/Akt/HIF-1alpha signaling pathway. Oncol Rep.

[15] Ferrick DA, Neilson A, Beeson C (2008). Advances in measuring cellular bioenergetics using extracellular flux. Drug Discov Today.

[16] Harris PS, Hansen RM, Gray ME, Massoud OI, McGuire BM, Shoreibah MG (2019). Hepatocellular carcinoma surveillance: an evidence-based approach. World J Gastroenterol.

[17] Chen D, Li Z, Cheng Q, Wang Y, Qian L, Gao J (2019). Genetic alterations and expression of PTEN and its relationship with cancer stem cell markers to investigate pathogenesis and to evaluate prognosis in hepatocellular carcinoma. J Clin Pathol.

[18] Khalid A, Hussain T, Manzoor S, Saalim M, Khaliq S (2017). PTEN: a potential prognostic marker in virus-induced hepatocellular carcinoma. Tumour Biol.

[19] Fu X, Wen H, Jing L, Yang Y, Wang W, Liang X (2017). MicroRNA-155-5p promotes hepatocellular carcinoma progression by suppressing PTEN through the PI3K/Akt pathway. Cancer Sci.

[20] Li Y, Ye Y, Feng B, Qi Y (2017). Long noncoding RNA lncARSR promotes doxorubicin resistance in hepatocellular carcinoma via modulating PTEN-PI3K/Akt pathway. J Cell Biochem.

[21] Han Y, Chen M, Wang A, Fan X (2019). STAT3-induced upregulation of lncRNA CASC11 promotes the cell migration, invasion and epithelial-mesenchymal transition in hepatocellular carcinoma by epigenetically silencing PTEN and activating PI3K/AKT signaling pathway. Biochem Biophys Res Commun.

[22] Lin H, Huang ZP, Liu J, Qiu Y, Tao YP, Wang MC (2018). MiR-494-3p promotes PI3K/AKT pathway hyperactivation and human hepatocellular carcinoma progression by targeting PTEN. Sci Rep.

[23] Kim J, Jung KH, Choi JG (2020). Artemisiae Iwayomogii Herba inhibits growth, motility, and the PI3K/AKT/mTOR signaling pathway in hepatocellular carcinoma cells. Planta Med.

[24] Lee M, Ko H, Yun M (2018). Cancer metabolism as a mechanism of treatment resistance and potential therapeutic target in hepatocellular carcinoma. Yonsei Med J.

[25] Lee M, Yoon JH (2015). Metabolic interplay between glycolysis and mitochondrial oxidation: the reverse Warburg effect and its therapeutic implication. World J Biol Chem.

[26] Shang RZ, Qu SB, Wang DS (2016). Reprogramming of glucose metabolism in hepatocellular carcinoma: Progress and prospects. World J Gastroenterol.

[27] Beyoglu D, Idle JR (2013). The metabolomic window into hepatobiliary disease. J Hepatol.

[28] Wang Y, Lin B, Li H, Lan L, Yu H, Wu S (2017). Galangin suppresses hepatocellular carcinoma cell proliferation by reversing the Warburg effect. Biomed Pharmacother.

[29] Liberti MV, Locasale JW (2016). The Warburg effect: how does it benefit cancer cells?. Trends Biochem Sci.

[30] Spencer NY, Stanton RC (2019). The Warburg effect, lactate, and nearly a century of trying to cure cancer. Semin Nephrol.

[31] Garcia-Cao I, Song MS, Hobbs RM, Laurent G, Giorgi C, de Boer VC (2012). Systemic elevation of PTEN induces a tumor-suppressive metabolic state. Cell..

[32] Li Y, He L, Zeng N, Sahu D, Cadenas E, Shearn C (2013). Phosphatase and tensin homolog deleted on chromosome 10 (PTEN) signaling regulates mitochondrial biogenesis and respiration via estrogen-related receptor alpha (ERRalpha). J Biol Chem.

[33] Bao L, Li X, Lin Z (2019). PTEN overexpression promotes glioblastoma death through triggering mitochondrial division and inactivating the Akt pathway. J Recept Signal Transduct Res.

[34] Zhang J, Li L, Peng Y, Chen Y, Lv X, Li S (1865). Surface chemistry induces mitochondria-mediated apoptosis of breast cancer cells via PTEN/PI3K/AKT signaling pathway. Biochim Biophys Acta, Mol Cell Res.

