# Correction to: Loss of PTEN expression is associated with PI3K pathway-dependent metabolic reprogramming in hepatocellular carcinoma

**DOI:** 10.1186/s12964-021-00745-8

**Published:** 2021-05-12

**Authors:** Chuanzong Zhao, Ben Wang, Enyu Liu, Zongli Zhang

**Affiliations:** 1 Department of General Surgery, Qilu Hospital of Shandong University, No. 107, Wenhua West Road, Lixia District, Jinan, 250012 Shandong Province People’s Republic of China; 2 Key Laboratory for Experimental Teratology of the Ministry of Education, Department of Pathology, School of Medicine, Shandong University, Jinan, 250012 People’s Republic of China

## Correction to: Cell Communication and Signaling (2020) 18:131 10.1186/s12964-020-00622-w

Following publication of the original article [1], the authors identified the following errors.

- In the first paragraph of the **Results/PTEN is downregulated in HCC and correlates with poor prognosis of HCC** section, “tumor classification and metastasis” should be replaced with “tumor size, classification and metastasis”.
- In the **Results/Overexpression of PTEN inhibits Warburg effect and maintains mitochondrial function in HCC cells** section, “a decreased proportion of red to green fluorescence” should be revised to “increased proportion of red to green fluorescence and recovered mitochondrial function”.
- In the legend of Fig. 3 “Ratio of red/green fluorescence intensity” should be changed to “Mitochondrial membrane potential ΔΨ”.

Further to this, the following change have been made to the Figs. 1, 2, 3, and 4.

- Figure 1: A and B, group typesetting name is inverted. The correct sequence is from left to right: adjacent to cancer, liver cancer, and also marked with PTEN and PI3K
- Figure 3: C, vector NC and OE PTEN group image typesetting and statistical errors, I revised the image typesetting
- Figure 4: the statistics of averctor NC and OE PTEN group are wrong. I revised the picture layout again

The updated figures and captions, as well as tables are given below:

**Fig. 1:**
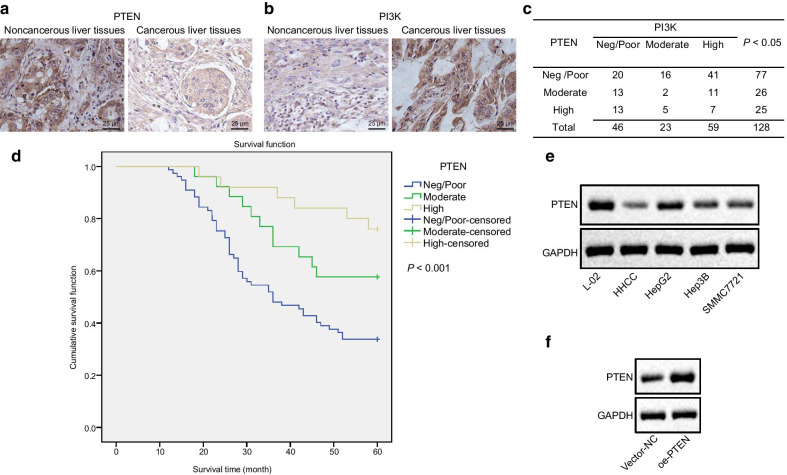
PTEN is downregulated in HCC and its loss predicts worse prognosis in HCC patients. **a** and **b**, Immunocytochemistry staining of PTEN (**a**) and PI3K (**b**) proteins in cancerous liver tissues (*n* = 128) and adjacent noncancerous liver tissues (*n* = 43) (× 400). *p < 0.05 compared with adjacent noncancerous liver tissues by unpaired *t*-test. **c**, Spearman’s correlation analysis of PTEN expression with PI3K expression in HCC tissues (*n* = 128). **d**, Kaplan–Meier curves are plotted to show overall survival of 128 HCC patients according to PTEN expression. **e**, Representative Western blots of PTEN protein in L-02, HepG2, Hep3B, SMMC7721, and HHCC cell lines, normalized to GAPDH. *p < 0.05 compared with L-02 cell lines. **f**, Verification of HHCC cells with lentiviral transduction of PTEN by Western blot analysis, normalized to GAPDH. HHCC cells were transduced with a lentiviral vector encoding full-length human PTEN (oe-PTEN), with those cells infected with a lentiviral vector harboring empty expression vector as controls (vector-NC). *p < 0.05 compared with vector-NC. Data are shown as mean ± standard deviation of three technical replicates

**Fig. 2:**
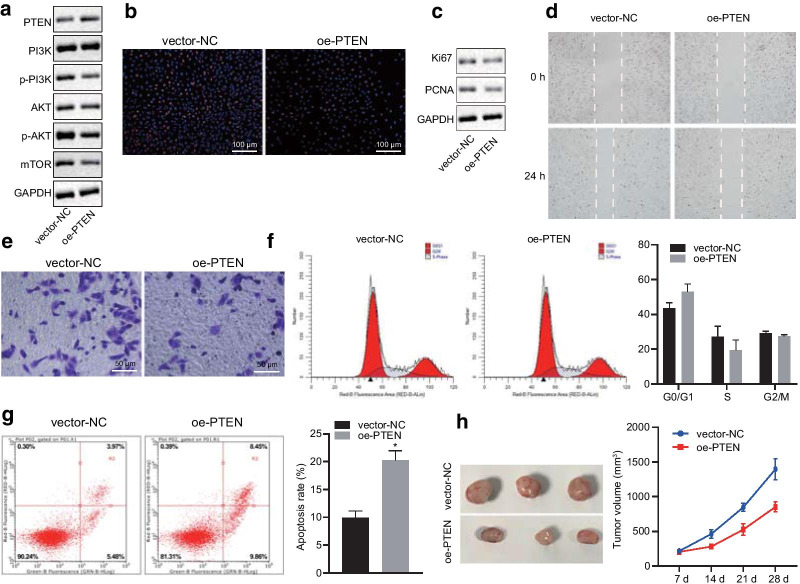
PTEN regulates the development of HCC in vitro and in vivo by inhibiting the activation of PI3K/Akt pathway. HHCC cells were transduced with a lentiviral vector encoding full-length human PTEN (oe-PTEN), with those cells infected with a lentiviral vector harboring empty expression vector as controls (vector-NC). **a**, Representative Western blots of PI3K, Akt, and mTOR proteins and their quantitation in HHCC cells, normalized to GAPDH. **b**, EdU-stained cells were captured (× 200) to reflect HHCC cell proliferation. **c**, Representative Western blots of proliferation markers Ki67 and PCNA and their quantitation in HHCC cells, normalized to GAPDH. **d**, Wound closure was monitored to measure HHCC cell migration (24 h after scratch). **e**, HHCC cells invading from Matrigel-coated the upper transwell chamber into the lower one. **f**, Flow cytometric analysis of PI staining was performed to examine the distribution of HHCC cells throughout G0/G1, S, and G2/M phases of the cell cycle. **g**, Flow cytometric analysis of Annexin V/PI double staining was performed to determine HHCC cell apoptosis. **h**, Representative HCC xenograft tumors and the growth of HCC xenograft tumor measured every 7 days in nude mice (*n* = 6). *p < 0.05 compared with vector-NC by unpaired *t*-test. Data are shown as mean ± standard deviation of three technical replicates

**Fig. 3:**
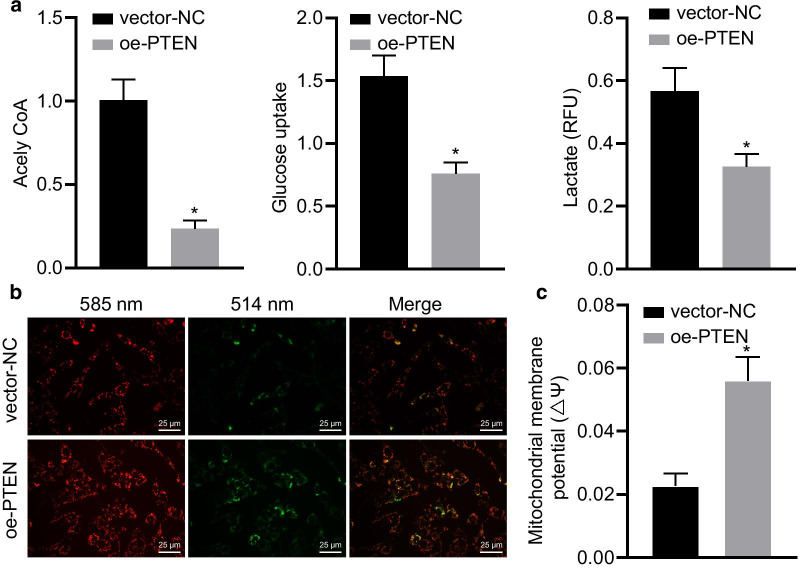
PTEN inhibits Warburg effect and maintains mitochondrial function in HCC cells. HHCC cells were transduced with a lentiviral vector encoding full-length human PTEN (oe-PTEN), with those cells infected with a lentiviral vector harboring empty expression vector as controls (vector-NC). **a**, Measurements of acetyl-CoA synthesis, glucose uptake, and lactate production in HHCC cells. **b**, JC-1 staining was performed to examine the MMP in HHCC cells (× 400). Red stains at 585 nm indicate JC-1 aggregates in intact mitochondria, and green stains at 514 nm indicate JC-1 monomer in apoptotic cells with depolarization of MMP. **c**, Mitochondrial membrane potential ΔΨ was calculated by flow cytometric analysis in HHCC cells. *p < 0.05 compared with vector-NC by unpaired *t*-test. Data are shown as mean ± standard deviation of three technical replicates

**Fig. 4:**
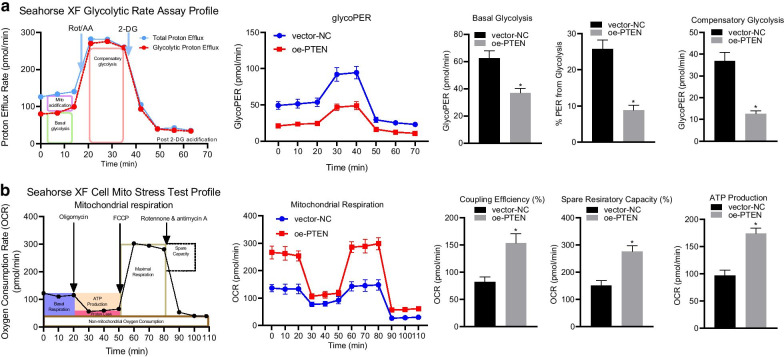
PTEN reduces ECAR and enhances OCR in HCC cells. HHCC cells were transduced with a lentiviral vector encoding full-length human PTEN (oe-PTEN), with those cells infected with a lentiviral vector harboring empty expression vector as controls (vector-NC). At first, the ECAR and OCR were measured under basal condition. Next, the ECAR was measured in the presence of rotenone plus the mitochondrial complex III inhibitor antimycin A (Rote/AA) and the glycolytic inhibitor 2-DG at indicated time points. The OCR was measured in the presence of oligomycin, the reversible inhibitor of oxidative phosphorylation FCCP (p-trifluoromethoxy carbonyl cyanide phenylhydrazone), and the Rote/AA at indicated time points. OCR is expressed as pmols/minute and ECAR as mpH/minute. **a**, ECAR under basal glycolysis, %ECAR from basal glycolysis, and the ECAR of compensatory glycolysis were measured by Seahorse Bioscience XF24 analyzer in HHCC cells. **b**, basal OCR, ATP-linked respiration, maximal OCR, and spare respiratory capacity were measured by Seahorse Bioscience XF24 analyzer in HHCC cells. Data in the upper (**a** and **b**) were compared using unpaired *t*-test and in the lower (**a** and **b**) by repeated measures ANOVA with Bonferroni corrections. *p < 0.05 compared with vector-NC. Data are shown as mean ± standard deviation of three technical replicates
